# Postoperative Weight-Bearing, Range-of-Motion Protocols and Knee Biomechanics After Concomitant Posterolateral Meniscal Root Repair with ACL Reconstruction: A Systematic Review

**DOI:** 10.3390/jcm15020542

**Published:** 2026-01-09

**Authors:** Thibaut Noailles, Julien Behr, Nicolas Bouguennec, Loïc Geffroy, César Tourtoulou, Alain Meyer

**Affiliations:** 1 Department of Orthopaedic Surgery, Polyclinique de Bordeaux Nord, 33 rue du Docteur Finlay, 33000 Bordeaux, France; noaillesthibaut@yahoo.fr; 2 Department of Orthopaedic Surgery, University Hospital of Nantes, 1 Place Alexandre Ricordeau, 44000 Nantes, France; 3 Department of Orthopaedic Surgery, Clinique du Sport, 4 rue Georges-Nègrevergne, 33700 Mérignac, France; nbouguennec@gmail.com; 4 Department of Orthopaedic Surgery, Polyclinique de l’Atlantique, Avenue Claude-Bernard, 44819 Saint-Herblain Cedex, France; geffroyloic@hotmail.fr; 5 Department of Orthopaedic Surgery, Clinique Aguiléra, 21 rue de l’Estagnas, 64200 Biarritz, France; cesar.tourtoulou@orange.fr; 6 Department of Orthopaedic Surgery, Clinique du Sport, 36 Boulevard Saint Marcel, 75005 Paris, France; dr.meyer@chirurgiedusport.com

**Keywords:** posterolateral meniscal root, ACL reconstruction, rehabilitation, weight-bearing, range of motion, systematic review, knee joint biomechanics

## Abstract

**Background/Objectives**: Meniscal root tears, particularly those of the posterolateral root, are frequently associated with anterior cruciate ligament (ACL) injuries and significantly alter load distribution and knee stability. Surgical repair of the posterolateral meniscal root (PLMR) aims to restore normal biomechanics; however, postoperative rehabilitation strategies remain heterogeneous. The objective of this systematic review was to describe and analyze postoperative weight-bearing (WB) and range-of-motion (ROM) protocols following concomitant PLMR repair and anterior cruciate ligament reconstruction (ACLR), integrating both clinical and biomechanical perspectives. **Methods**: This systematic review followed PRISMA guidelines and analyzed biomechanical and clinical studies assessing postoperative WB and ROM management following PLMR repair combined with ACLR. **Results**: Eleven studies were included, describing heterogeneous postoperative rehabilitation protocols for WB and ROM following posterolateral meniscal root repair with ACLR. Biomechanical data consistently showed that root section increased tibial internal rotation and contact pressure on the lateral tibial plateau, whereas repair restored near-native load sharing. Clinically, most authors recommended non-weight-bearing or toe-touch loading for 4–6 weeks and flexion limited to 0–90° during early rehabilitation. Gradual progression to full loading and motion between 8 and 12 weeks was the most consistent strategy. **Conclusions**: Although the current evidence is limited and mainly based on low-level studies, available data suggest that a cautious and progressive rehabilitation protocol after PLMR repair with ACLR early controlled motion and delayed full loading may optimize repair healing while protecting graft integrity.

## 1. Introduction

Meniscal root tears, which have been reported to accompany anterior cruciate ligament injuries in 10–15% of cases, are increasingly recognized as key secondary stabilizers of the knee. These tears significantly alter knee biomechanics by disrupting hoop stress and increasing tibiofemoral contact pressure [1,2,3,4].

Among these, PLMR tears are particularly associated with ACL injuries, leading to increased rotational instability and greater stress on the reconstructed graft [5,6,7,8].

PLR tears are defined as radial tears within 1 cm of the PL root or an avulsion of the insertion [9]. Magnetic resonance imaging (MRI) signs indicative of meniscal root abnormality include a radial tear of the meniscal root (on axial imaging), a vertical linear defect in the meniscal root (truncation sign on coronal imaging), meniscal extrusion > 3 mm outside the peripheral margin of the joint (on coronal imaging), and increased signal within the meniscal root (ghost sign on sagittal sequences) [10,11,12].

Repair of the PLMR has been shown to restore load distribution and knee stability, but postoperative rehabilitation protocols remain heterogeneous across the literature [13].

The balance between protecting biological healing and preventing stiffness is crucial during the early postoperative phase. Despite the growing number of studies on meniscal root repair, there is still no consensus on the optimal timing for weight-bearing and ROM progression, especially when combined with ACLR [14,15].

Postoperative rehabilitation following combined PLMR repair and ACLR plays a fundamental role in protecting early biological healing. Although ACL rehabilitation principles are well standardized in the orthopedic literature, postoperative protocols specific to meniscal root fixation remain largely empirical and inconsistently reported [13,16,17,18]. Most clinical series recommend cautious progression during the early postoperative period, including non-weight-bearing or toe-touch loading for the first 4–6 weeks, followed by gradual advancement toward full weight-bearing between 8 and 10 weeks. Early range-of-motion is generally limited to controlled flexion within 0–90° during the first month to reduce posterior compartment pressure while simultaneously maintaining full extension and allowing early quadriceps recruitment. Flexion beyond 90° and full motion are typically reintroduced between 8 and 12 weeks, although no comparative clinical or randomized data exist to validate superiority of early or delayed motion strategies.

A limited number of observational studies have reported satisfactory outcomes using progressive, criterion-based postoperative rehabilitation, but the evidence remains fragmented, retrospective in nature, and difficult to generalize due to heterogeneous surgical techniques and inconsistent reporting of rehabilitation parameters [13,16,17,18]. Most current recommendations therefore rely on expert opinion rather than high-level comparative data, highlighting a substantial knowledge gap regarding safe progression of loading and motion following concomitant PLMR repair and ACLR.

These limitations underscore the need for a synthesized research perspective integrating both biomechanical evidence and clinical postoperative practices. Understanding early graft–root interaction, the biomechanical consequences of premature loading, and the physiological implications of motion restrictions is essential to optimize functional recovery and long-term joint preservation.

Given the absence of PLMR-specific postoperative guidelines and the heterogeneity observed across current reports, a systematic evaluation of rehabilitation principles is required to clarify whether gradual loading and controlled motion safely support healing after concomitant PLMR repair and ACLR. Previous rehabilitation reviews and expert consensus statements have already suggested that a criterion-based and progressive postoperative approach may be clinically appropriate after meniscal repair and ACLR [13,14,15], which provides a scientific rationale for the research direction and the formulation of our hypothesis.

**Hypothesis:** We hypothesized that most studies would support a cautious and progressive rehabilitation program emphasizing restricted early loading and controlled motion in order to protect meniscal fixation and ACL graft integration during early biological healing.

## 2. Materials and Methods

### 2.1. Search Strategy and Study Design

This review followed Preferred Reporting Items for Systematic Reviews and Meta-Analyses (PRISMA) 2020 guidelines and the Cochrane Handbook for Systematic Reviews of Interventions [19,20] (Table S1). Databases searched included PubMed, MEDLINE, Embase, CINAHL, and the Cochrane Library through August 2025, using combinations: (“meniscus root” OR “meniscal root” OR “posterolateral root” OR “posterior horn” OR “radial tear” OR “root avulsion”) AND (“ACL reconstruction” OR “anterior cruciate ligament reconstruction” OR “ACL injury”) AND (“rehabilitation” OR “physical therapy” OR “weight-bearing” OR “range of motion” OR “mobilization”).

As no primary patient enrollment or interventional data collection occurred, no sample size calculation or statistical power analysis was applicable. Risk of bias was inferred qualitatively based on study methodology, clarity of rehabilitation descriptions, and variability in follow-up. Biomechanical investigations were evaluated for reproducibility, loading conditions, fixation stability, and experimental controls.

Overall, the present work should be characterized as a descriptive systematic review, combining qualitative synthesis of clinical series and biomechanical experiments rather than a quantitative meta-analysis.

### 2.2. Eligibility Criteria

Included studies reported biomechanical and clinical outcomes related to postoperative rehabilitation after PLMR repair with ACLR. Case reports, animal studies, technical notes, and non-English articles were excluded.

For this review, we specifically focused on studies reporting postoperative rehabilitation or biomechanical outcomes for lateral or posterolateral meniscal root tears treated concomitantly with ACLR. When articles included both medial and lateral root tears, data were screened to identify whether outcomes for lateral or PLMR lesions were reported separately. If separate reporting was available, only the subgroup corresponding to lateral or PLMR tears was extracted. When outcomes for medial and lateral roots were irreversibly pooled, the study was either excluded from detailed analysis or used only to support broader qualitative statements. Similarly, in studies reporting multiple meniscal pathologies, only those cases in which the rehabilitation protocol and outcomes could be clearly attributed to lateral or PLMR repair with ACLR were retained for synthesis. This approach was chosen to preserve the specificity and purity of the evidence base regarding PLMR repair combined with ACLR.

For anatomical consistency, the term “posterolateral meniscal root” was considered equivalent to “lateral meniscus posterior root” (LMPR), as both describe the posterior tibial attachment of the lateral meniscus. Throughout the literature, these terms are used interchangeably by different authors and surgical teams to identify the same anatomical structure. Studies explicitly reporting LMPR repair in combination with ACLR were therefore considered relevant and included in the synthesis, provided that the rehabilitation protocol or biomechanical outcomes could be clearly attributed to the lateral root.

### 2.3. Study Selection and Data Extraction

Two reviewers (T.N., A.H.) independently screened titles, abstracts, and full texts. Data on study design, population, technique, rehabilitation timeline (weight-bearing and ROM), and outcomes were extracted and validated by consensus with a third reviewer (J.B.).

### 2.4. Quality Assessment

Clinical studies were assessed using the modified Coleman Methodology Score (mCMS), while biomechanical studies were evaluated based on adapted experimental quality criteria derived from previously published cadaveric validation frameworks. Risk of bias was assessed in accordance with PRISMA Item 11. For clinical studies, two reviewers independently evaluated methodological quality using the modified Coleman Methodology Score, with particular attention to study design, inclusion criteria, clarity of rehabilitation reporting, duration and completeness of follow-up, and consistency of outcome measures. Potential sources of bias included selection bias (retrospective design, non-randomized cohorts), confounding (heterogeneous surgical techniques or concomitant procedures), and reporting bias (incomplete description of rehabilitation or complications). For biomechanical studies, risk of bias was inferred from specimen selection, standardization of testing protocols, control of loading conditions, reproducibility, and adequacy of statistical analysis. Disagreements were resolved by consensus with a third reviewer.

### 2.5. Data Synthesis

**Figure 1:**
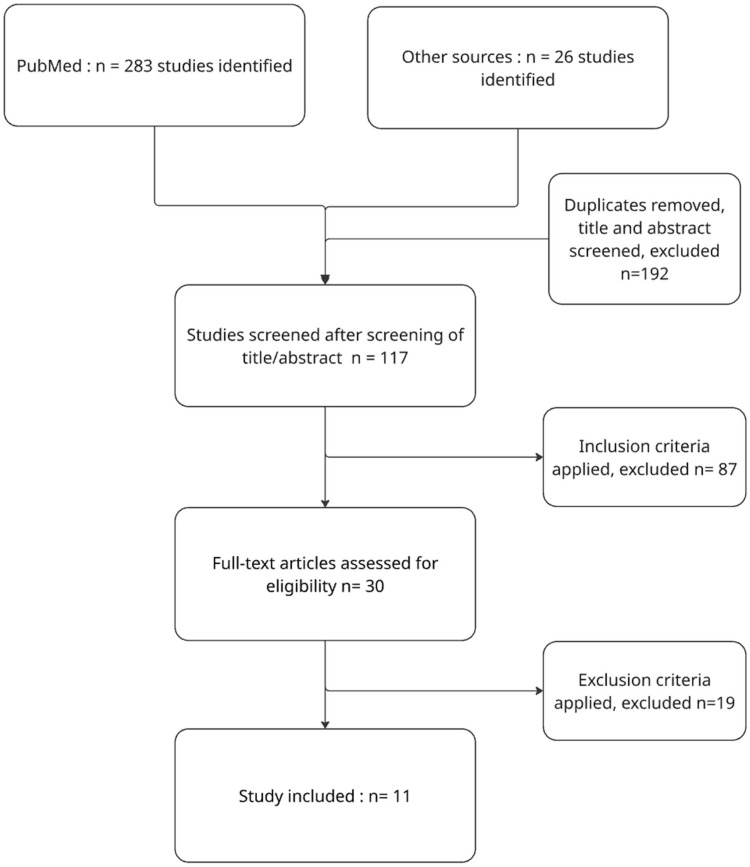
PRISMA 2020 Flow Diagram illustrating the study selection process.

Due to heterogeneity, findings were summarized qualitatively. The study selection process is presented in Figure 1, and rehabilitation protocols in Table 1.

## 3. Results

### 3.1. Study Selection

The initial search identified 309 records. After duplicate removal and screening, 117 full-text articles were assessed for eligibility. Among these, 56 studies analyzed range-of-motion protocols and 61 addressed weight-bearing rehabilitation. After applying inclusion criteria, 11 studies were retained for qualitative analysis. The PRISMA flow diagram summarizing this process is shown in Figure 1.

### 3.2. Methodological Quality of Included Studies

Overall, the methodological quality of clinical studies was fair to good (mCMS 60–68/100). Biomechanical investigations demonstrated consistent experimental rigor, with scores ranging from 6 to 9 out of 10, reflecting adequate control, reproducibility, and statistical analysis (Table 2). The fair mCMS scores (60–68/100) observed for clinical studies primarily reflect the inherent limitations of retrospective designs. Most series included non-randomized cohorts with potential selection bias, heterogeneous surgical techniques and rehabilitation protocols, and relatively short follow-up periods, often shorter than 2 years. These factors increase the risk of confounding and under-reporting of late failures or degenerative changes, and they limit the strength of causal inferences that can be drawn from the available data. Consequently, while the reported outcomes are encouraging, they should be interpreted within the context of these methodological constraints.

### 3.3. Biomechanical Findings

The available biomechanical literature addressing posterolateral meniscal root tears and their repair remains limited in number and scope. Existing cadaveric studies consistently demonstrate that sectioning of the PLMR results in increased internal tibial rotation, anterior tibial translation, and elevated contact pressure on the lateral tibial plateau, particularly between 60° and 90° of flexion. Loss of the meniscal root attachment markedly reduces hoop tension and alters the distribution of load across the tibiofemoral joint, leading to focal overpressure in the posterior compartment [21,22]. Repair of the root, particularly through transosseous pull-out techniques, restores near-native contact mechanics and rotational stability in most cadaveric models [6,23]. Combined PLMR repair and ACLR better re-establishes anterior and rotational stability than isolated ACLR, without evidence of overconstraint or abnormal lateral compartment loading [5,6,7]. Nevertheless, the biomechanical evidence base is scarce, relying on small cadaveric samples, heterogeneous methodologies, and absence of long-term in vivo validation. Despite these limitations, current data support that anatomic PLMR repair restores native load distribution and joint stability when performed in conjunction with ACLR.

### 3.4. Weight-Bearing Protocols

The literature on postoperative weight-bearing after PLMR repair combined with ACLR is remarkably limited, with only a handful of clinical series providing explicit protocols [13,16,17]. Across these studies, the rehabilitation approach is cautious and largely empirical, as no comparative or randomized data exist. Most authors recommend non-weight-bearing or toe-touch WB for the first 4–6 weeks, followed by gradual progression to full loading between 8 and 10 weeks. Shekhar et al. [13] applied NWB for 4 weeks with advancement based on clinical tolerance, while de Faria et al. [18] maintained unloading for 6 weeks before WB as tolerated. Langhans et al. [16] similarly prescribed toe-touch (<10 kg) loading for 4 weeks, achieving full WB by 8–10 weeks. Pan et al. [17] allowed partial WB from week 2, reflecting early ACLR-style rehabilitation. No study directly compared early versus delayed WB after PLMR repair, and none demonstrated superior outcomes with early full loading. Biomechanical findings indicate that premature compression increases lateral tibial contact pressure and could jeopardize both the meniscal fixation and ACL graft [6,7]. Overall, the evidence remains scarce and low-level, preventing firm recommendations. Current practice favors restricted or partial WB for 4–6 weeks, followed by criterion-based progression, though these protocols are based on expert judgment rather than high-quality comparative data.

### 3.5. Range-of-Motion Protocols

Evidence regarding ROM management after PLMR repair with ACLR is similarly limited and heterogeneous. Only a few clinical series describe their postoperative motion restrictions in detail [13,16,17,18]. Most protocols restrict knee flexion to 0–90° for the first 4 weeks, promote early restoration of full extension, and introduce gentle passive motion during the initial phase. Flexion beyond 90° is typically delayed until week 6, while deep loaded flexion and squatting are avoided for 3–4 months. Between 4 and 6 weeks, flexion is gradually increased to 120°, provided that swelling and pain are minimal, with full motion restored by 10–12 weeks in most reports. No clinical study to date has compared early versus delayed motion protocols after PLMR repair. However, limited observational data suggest that controlled early motion aids quadriceps activation and reduces stiffness without compromising repair integrity [17,18]. Importantly, no repair failure or extension deficit related to early motion has been reported. Taken together, the current evidence base is very weak, derived from small retrospective cohorts and lacking standardization. A conservative but progressive ROM regimen remains the most accepted strategy early extension and controlled flexion recovery during the first 6 weeks, with return to full motion by 3 months though this approach is founded on expert consensus and low-level evidence rather than comparative clinical trials.

Across the biomechanical and limited clinical evidence, PLMR injury increases rotational instability and lateral compartment loading, whereas anatomic repair combined with ACLR restores near-native joint kinematics and contact pressures [5,6,7]. Nevertheless, the rehabilitation literature remains notably sparse, with only a few small series reporting detailed protocols. Most recommend 4–6 weeks of restricted WB and flexion limitation, followed by gradual reloading and motion recovery between 6 and 12 weeks, achieving satisfactory healing and function but lacking comparative validation. The overall level of evidence is low (Level IV–V), and further prospective, controlled studies are needed to define safe and efficient rehabilitation strategies after combined PLMR and ACLR.

## 4. Discussion

This systematic review highlights the limited and heterogeneous evidence available regarding postoperative rehabilitation following PLMR repair combined with ACLR. Despite the growing number of technical reports and expert recommendations, there remains a notable lack of high-level comparative data to guide postoperative management. The majority of published literature consists of biomechanical cadaveric studies, low-level clinical series (Level III–IV), and technical notes, while systematic reviews and consensus statements—such as those from ESSKA–AOSSM collaborations provide guidance largely extrapolated from medial root or general meniscal repair protocols rather than PLMR-specific evidence.

Across published biomechanical investigations, the rationale for early protection is consistently supported. Premature compressive loading or deep flexion increases posterior compartment pressure and alters hoop stress transmission across the lateral tibial plateau, potentially jeopardizing root fixation or early biological integration. Controlled motion limited to 0–90° during the initial weeks allows quadriceps activation and mobility while minimizing rotational stress and contact overload, which provides a physiological explanation for delayed progression to full loading. This biomechanical interpretation aligns with the gradual rehabilitation strategies reported across clinical series and supports cautious early progression following concomitant PLMR repair and ACLR.

Despite the overall convergence toward progressive rehabilitation, the evidence remains limited by methodological heterogeneity. Most clinical studies were retrospective, used different fixation techniques, and reported rehabilitation parameters imprecisely, with short follow-up periods that may underestimate late failures or degenerative changes. The fair mCMS scores (60–68/100) observed across these investigations reflect the absence of randomization, potential selection bias, and confounding due to multiple concomitant procedures. As a result, the level of certainty remains moderate, and current recommendations should be interpreted as principles of best practice rather than definitive protocols validated by comparative trials.

### 4.1. Biomechanical Considerations

Biomechanical investigations consistently show that sectioning of the PLMR disrupts tibiofemoral congruence, increases lateral compartment loading, and induces anterior translation and internal tibial rotation [4,5,7]. Repair, especially using anatomic transosseous pull-out fixation, restores hoop tension and normalizes contact pressure across the lateral tibial plateau [6,23]. These findings support protective rehabilitation strategies during the early healing phase, particularly when combined with ACLR, where excessive early loading could compromise both meniscal fixation and graft incorporation. However, biomechanical data are derived from small, controlled laboratory models and lack validation in vivo, underscoring the need for translational and clinical correlation.

These biomechanical insights provide a rationale for cautious rehabilitation protocols described in the following sections.

### 4.2. Weight-Bearing Management

Among clinical studies, progressive loading after 4–6 weeks remains the most common recommendation [13,16,18]. Early unrestricted weight-bearing has not demonstrated superior outcomes and may increase the risk of shear stress at the root–bone interface, potentially jeopardizing repair integrity [6,7]. In contrast, delayed full loading (8–10 weeks) appears to favor biological healing, particularly when progression is guided by pain, swelling, and quadriceps control [13,18].

Comparable postoperative strategies have been proposed in technical descriptions by Stokes et al. [25], Matassi [26] et al. and Laddha et al. [27], all of whom advocate restricted or toe-touch weight-bearing during the first 4–6 weeks, followed by gradual progression to full loading by approximately 8–10 weeks. These recommendations, although derived from expert experience rather than comparative trials, are consistent with the conservative approach observed across clinical series.

Nonetheless, no randomized study has directly compared early versus delayed loading after PLMR repair. Much of the current guidance originates from expert opinion, technical notes, and consensus statements, such as the EU–US Meniscus Rehabilitation Consensus [15], which advocates criterion-based progression but acknowledges the absence of PLMR-specific validation. Overall, available evidence supports a cautious, progressive approach, prioritizing biological protection during the first 4–6 weeks and individualized progression thereafter.

### 4.3. Range-of-Motion Progression

ROM management follows a similarly conservative pattern across available reports. Most authors limit flexion to 0–90° during the first 4 weeks, encouraging early restoration of full extension and gradual flexion progression thereafter [13,16,18]. Full flexion and squatting are typically delayed until 3–4 months, a timeframe consistent with recommendations from meniscal repair reviews and recent rehabilitation consensus statements [14,15].

Comparable timelines and progression strategies are also described in technical protocols proposed by Stokes et al. [25], Matassi et al. [26], and Laddha et al. [27]., all of whom recommend controlled motion within 90° during the initial 4–6 weeksand deferral of deep loaded flexion beyond 120° until after the third postoperative month. Although these reports are based on expert experience rather than comparative data, they reinforce a consistent rationale across the literature: to protect the posterior root fixation and the reconstructed ACL graft while minimizing the risk of stiffness and loss of motion.

While controlled early motion (within 90°) may facilitate quadriceps activation and joint mobility, no comparative study has yet assessed its safety or superiority after PLMR repair [13,18]. Consequently, current ROM protocols remain empirical and largely extrapolated from ACLR rehabilitation paradigms, supplemented by expert-based recommendations and consensus guidance rather than supported by dedicated PLMR-specific evidence.

### 4.4. Clinical Implications

Taken together, the existing data suggest that early protection followed by gradual reactivation represents a safe and effective rehabilitation strategy after PLMR repair with ACLR. A criterion-based progression taking into account pain, effusion, range of motion, and quadriceps control—is recommended. However, clinicians should be aware that these recommendations stem primarily from low-level evidence and expert consensus, and therefore require individualized clinical judgment.

### 4.5. Limitations and Future Directions

The present review underlines the paucity and methodological heterogeneity of the available literature. Most included studies are retrospective series with small sample sizes, often combining various meniscal tear patterns or surgical techniques, limiting generalizability. No prospective randomized trial has yet compared different rehabilitation strategies after PLMR repair, and no standardized outcome measure exists for evaluating postoperative recovery in this population.

In addition, systematic reviews and consensus statements, while valuable, tend to aggregate medial and lateral root data or focus on isolated ACLR rehabilitation, making it difficult to draw PLMR-specific conclusions. Despite the biomechanical relevance of PLMR integrity, only a handful of small clinical series provide rehabilitation details, highlighting the striking paucity of evidence in this field.

Future studies should therefore aim to establish standardized rehabilitation protocols, ideally through prospective multicenter cohorts or randomized controlled trials assessing different timelines of loading and motion.

## 5. Conclusions

A cautious and progressive rehabilitation program following concomitant PLMR repair and ACLR appears justified based on current biomechanical evidence and early clinical experience. Restricted loading during the first 4–6 weeks, combined with controlled motion within 0–90°, protects graft integrity and meniscal fixation while allowing early mobility. Gradual progression toward full loading and range of motion between 8 and 12 weeks reflects a balanced approach between biological protection and functional recovery. However, existing studies remain small, heterogeneous, and mainly retrospective, preventing definitive recommendations. Future prospective and comparative investigations are needed to standardize rehabilitation timelines, confirm safety profiles, and determine long-term joint preservation outcomes after this combined procedure.

## Figures and Tables

**Table 1:** Summary of postoperative rehabilitation protocols reported in clinical studies (weight-bearing and ROM timelines, progression criteria, outcomes).

Author(s)	Year	Type	Weight-Bearing	Range of Motion	Key Points
Espejo-Reina et al. [21]	2022	Biomechanical (cadaveric)	–	Tested 0–90° flexion under cyclic load	Evaluated fixation strength under motion; supports limited early flexion to protect repair.
Forkel et al. [7]	2018	Biomechanical (cadaveric)	–	30–90° flexion tested	PLMR sectioning increased tibial rotation; repair restored stability in ACL-deficient knees.
Geeslin et al. [5]	2016	Biomechanical (cadaveric)	–	0–90° flexion	Combined PLMR repair + ACLR restored near-normal contact pressure.
Shybut et al. [4]	2015	Biomechanical (cadaveric)	–	15–90° ROM simulated pivot	Root tear increased rotational instability; emphasizes need for protection post-repair.
Abraham et al. [22]	2013	Biomechanical (cadaveric)	–	Pressure measured 0–90°	Root detachment increased compartmental load, justifying restricted flexion early on.
Tang et al. [6]	2019	Biomechanical (cadaveric)	–	0–90° tested	LMPR repair + ACLR restored anterior and rotational stability; excessive flexion increased load.
Lin et al. [23]	2013	Biomechanical (cadaveric)	–	Simulated 90–135° flexion	No gapping under deep flexion; confirmed stability of transosseous fixation.
Cuéllar et al. [24]	2015	Biomechanical (cadaveric)	–	0–90° ROM	Analyzed neurovascular safety by flexion angle; supports limiting deep flexion initially.
Shekhar et al. [13]	2022	Clinical	NWB 4 weeks → gradual WB	0–90° × 4 weeks → 120° × 6 weeks → full by 12 weeks	Progressive WB and ROM safe; good function, no rerupture.
Rocha de Faria et al. [18]	2022	Clinical	NWB 6 weeks → WB as tolerated	Passive ROM ≤ 90° × 4–6 weeks; full 10–12 weeks	Supports early extension and cautious flexion; low complication rate.
Langhans et al. [16]	2023	Clinical	NWB 4 weeks → full WB by 8–10 weeks	0–90° for 4 weeks, then gradual	Combined ACLR + PLMR repair; safe recovery with progressive protocol.

Abbreviations: NWB—non-weight-bearing; WB—weight-bearing; ROM—range of motion; ACLR—anterior cruciate ligament reconstruction; LMPR—lateral meniscus posterior root; PLMR—posterolateral meniscus root.

**Table 2:** Quality assessment of included studies.

Author(s)	Year	Study Type	Assessment Tool	Score/Criteria	Quality Rating	Comments
Shekhar et al. [13]	2022	Clinical	Modified Coleman Score	68/100	Good	Retrospective cohort; clearly defined rehab protocol; limited sample size.
Rocha de Faria et al. [18]	2022	Clinical	Modified Coleman Score	62/100	Fair	Multicenter study; consistent rehab reporting; short follow-up.
Langhans et al. [16]	2023	Clinical	Modified Coleman Score	60/100	Fair	Combined ACLR + PLMR repair; limited functional data.
Tang et al. [6]	2019	Biomechanical	Adapted Experimental Criteria	8/10	Good	Repeated trials, adequate fixation control.
Forkel et al. [7]	2018	Biomechanical	Adapted Experimental Criteria	9/10	Good	Clear protocol; appropriate statistical analysis.
Geeslin et al. [5]	2016	Biomechanical	Adapted Experimental Criteria	8/10	Good	Valid control group; realistic loading conditions.
Espejo-Reina et al. [21]	2022	Biomechanical	Adapted Experimental Criteria	7/10	Fair	Limited sample size; cyclic loading relevant.
Abraham et al. [22]	2013	Biomechanical	Adapted Experimental Criteria	7/10	Fair	Measured intra-tissue pressure; no mechanical fatigue test.
Lin et al. [23]	2013	Biomechanical	Adapted Experimental Criteria	8/10	Good	High testing precision; lacks clinical correlation.
Cuéllar et al. [24]	2015	Biomechanical	Adapted Experimental Criteria	6/10	Fair	Focused on neurovascular safety; not primary biomechanical endpoints.
Shybut et al. [4]	2015	Biomechanical	Adapted Experimental Criteria	8/10	Good	Robust pivot simulation model; adequate reproducibility.

Abbreviations: ACLR—anterior cruciate ligament reconstruction; PLMR—posterolateral meniscus root. Clinical studies were assessed using the modified Coleman Methodology Score (mCMS), while biomechanical studies were evaluated with adapted experimental quality criteria (10-point scale).

## Data Availability

All data supporting this review are available within the cited scientific literature.

## Supplementary Materials

The following supporting information can be downloaded at: https://www.mdpi.com/article/10.3390/jcm15020542/s1, Table S1. PRISMA Checklist.
