# Supplementary material for: Postoperative Weight-Bearing, Range-of-Motion Protocols and Knee Biomechanics After Concomitant Posterolateral Meniscal Root Repair with ACL Reconstruction: A Systematic Review
Source: J Clin Med. 2026 Jan 9;15(2):542. doi: 10.3390/jcm15020542 (PMC12842352; doi:10.3390/jcm15020542)
Supplement: Supplementary file 1 [file jcm-15-00542-s001.zip › jcm-4017067-supplementary.pdf]

Table S1. PRISMA Checklist.

Section / Topic	Item	PRISMA 2020 Recommendation	Location in manuscript
TITLE	1	Identify the report as a systematic review.	Title page: 'A Systematic Review'
ABSTRACT	2	Provide a structured summary including background, objectives, data sources, eligibility criteria, participants, interventions, study appraisal, synthesis methods, results, limitations, and conclusions.	Structured abstract (p.1)
INTRODUCTION	3	Describe the rationale for the review in the context of existing knowledge.	Introduction (p.2)
	4	Provide an explicit statement of the objectives or questions being addressed.	End of Introduction (p.2)
METHODS	5	Specify inclusion and exclusion criteria.	Methods – 'Eligibility Criteria' (p.2)
	6	Specify all information sources (databases, registers, etc.) and date last searched.	Methods – 'Search Strategy' (p.2)
	7	Present the full search strategy for at least one database.	Supplementary Table S1
	8	Specify the process for selecting studies (screening, eligibility, inclusion).	Methods – 'Study Selection' (p.2)
	9	Describe the data collection process.	Methods – 'Data Extraction' (p.2)
	10	List and define all variables for which data were sought.	Methods – 'Data Extraction' (p.2)
	11	Describe any methods used to assess risk of bias or quality of included studies.	Methods – 'Quality Assessment' (p.2)
	12	Specify the methods for data synthesis and handling of heterogeneity.	Methods – 'Data Synthesis' (p.3)
RESULTS	13	Describe the results of the search and selection process, ideally using a flow diagram.	Results – 'Study Selection' + Figure 1 (PRISMA flowchart)
	14	Provide characteristics of included studies.	Table 1 + Results summary (p.3
	15	Present risk of bias or quality assessments.	Table 2 – Quality Assessment (p.3)
	16	Present results of individual studies.	Results – 'Weight-Bearing Protocols' & 'ROM Protocols' (p.3)
	17	Present results of syntheses (narrative synthesis).	Results summary (p.3)
DISCUSSION	18	Summarize the main findings, including strength of evidence and limitations.	Discussion (p.4,5)
	19	Discuss limitations of the evidence and of the review process.	Discussion – 'Limitations' (p.5)
	20	Provide a general interpretation and implications for practice and research.	Conclusion (p.5,6)
OTHER INFORMATION	21	Registration and protocol (if available).	Not registered (mentioned in Methods)
	22	Support: Describe sources of financial or non-financial support.	End of manuscript – 'Funding: None'
	23	Competing interests.	End of manuscript – 'Conflict of Interest: None'
	24	Availability of data, code, and materials.	Supplementary materials statement
